# Investigating prostate cancer tumour–stroma interactions: clinical and biological insights from an evolutionary game

**DOI:** 10.1038/bjc.2011.517

**Published:** 2011-12-01

**Authors:** D Basanta, J G Scott, M N Fishman, G Ayala, S W Hayward, A R A Anderson

**Affiliations:** 1 Integrated Mathematical Oncology, H. Lee Moffitt Cancer Center and Research Institute, Tampa, FL 33612, USA; 2 Department of Radiation Oncology, H. Lee Moffitt Cancer Center and Research Institute, Tampa, FL 33612, USA; 3 Department of Genitourinary Oncology, H. Lee Moffitt Cancer Center and Research Institute, Tampa, FL 33612, USA; 4 Departments of Pathology, Immunology and Scott Department of Urology, Baylor College of Medicine, 1 Baylor Plz, Houston, TX 77030, USA; 5 Departments of Cancer Biology, Urologic Surgery and The Vanderbilt-Ingram Cancer Center, Vanderbilt University Medical Center, Nashville, TN 37232, USA

**Keywords:** prostate cancer, somatic evolution, evolutionary game theory, therapy, metastasis

## Abstract

**Background:**

Tumours are made up of a mixed population of different types of cells that include normal structures as well as ones associated with the malignancy, and there are multiple interactions between the malignant cells and the local microenvironment. These intercellular interactions, modulated by the microenvironment, effect tumour progression and represent a largely under-appreciated therapeutic target. We use observations of primary tumour biology from prostate cancer to extrapolate a mathematical model. Specifically, it has been observed that in prostate cancer three disparate cellular outcomes predominate: (i) the tumour remains well differentiated and clinically indolent – in this case the local stromal cells may act to restrain the growth of the cancer; (ii) early in its genesis the tumour acquires a highly malignant phenotype, growing rapidly and displacing the original stromal population (often referred to as small cell prostate cancer) – these less common aggressive tumours are relatively independent of the local microenvironment and (iii) the tumour co-opts the local stroma – taking on a classic stromagenic phenotype where interactions with the local microenvironment are critical to the cancer growth.

**Methods:**

We present an evolutionary game theoretical construct that models the influence of tumour–stroma interactions in driving these outcomes. We consider three characteristic and distinct cellular populations: stromal cells, tumour cells that are self-reliant in terms of microenvironmental factors and tumour cells that depend on the environment for resources, but can also co-opt stroma.

**Results:**

Using evolutionary game theory we explore a number of different scenarios that elucidate the impact of tumour–stromal interactions on the dynamics of prostate cancer growth and progression, and how different treatments in the metastatic setting can affect different types of tumours.

**Conclusion:**

The tumour microenvironment has a crucial role in selecting the traits of the tumour cells that will determine prostate cancer progression. Equally important treatments like hormone therapy affect the selection of these cancer phenotypes making it very important to understand how they impact prostate cancer's somatic evolution.

When detected early, prostate cancer is a largely curable entity. Once metastatic, like many cancers, cure becomes impossible and the only option left is a strategy of chronic disease management. Metastatic prostate cancer is initially managed with therapies aimed at ablating endogenous androgen production, a strategy that deprives cells of growth-signalling factors. In addition, bisphosphonates or RANK ligand inhibitors disrupt tumour–stroma interactions in the bone and are routinely considered as part of the standard of care for metastatic diseases (Gallo *et al*, 2011). Given a sufficiently long time interval, the emergence of resistance to these strategies is inevitable – for example, with hormone therapy resistance, this constitutes a situation called castrate resistance. Once resistance emerges, the disease becomes much more difficult to control, symptoms worsen and the life expectancy drastically shortens.

Carcinogenesis and cancer progression result from evolutionary processes in which the interactions between tumour cells, their environment and the surrounding stroma results in proliferation of cells from the genetically unstable tumour and, consequently, clinically malignant behaviour. The concept of microenvironmental changes associated with and potentially promoting expansion of the clones with phenotypes that define cancer dates back to observations by Galen in the second century (Reedy, 1975). Bone marrow-derived cells, widely distributed through tissues, have a complex range of roles including generation of immune and inflammatory responses, and contributing to fibrotic changes.

The role of specific cell types within this complex hierarchy is becoming better understood. Inflammatory cells have been shown to be involved in tumour promotion at many sites, including the prostate (De Marzo *et al*, 2007; Mantovani *et al*, 2008; Balkwill, 2009). This is likely due to the stimulation of persistent proliferative conditions in the presence of a mutagenic environment; some associated factors include the local activation of reactive oxygen species (Kundu and Surh, 2008; Schetter *et al*, 2010).

Fibroblasts and osteoclasts are also known to change in response to the presence of a tumour. In a series of papers in the 1980s, Schor and co-workers demonstrated that fibroblasts adjacent to carcinoma epithelium were fundamentally different from normal stroma and that these changes were implicated in neoplastic progression (Schor *et al*, 1987). These malignancy-associated changes occurred only in a subset of the resident fibroblasts (Schor and Schor, 1987; Schor *et al*, 1988). Specific references to tumour-associated or carcinoma-associated fibroblasts, myofibroblasts and reactive stroma have become abundant in literature from the 1970s onwards.

The relationship between osteoclasts and tumour cells in metastatic disease in the bone has been of recent interest, and much research has gone into understanding signalling cascades including many of the matrix metalloproteinases (Lynch *et al*, 2005), which seem to promote a vicious cycle of bone turnover and tumour promotion. Sadly, although these pathways are reasonably well understood, the clinical application of our knowledge in this realm has had little impact with the notable exception of cladronate in breast cancer – a case where a stromal-directed therapy, not an anticancer agent, has actually been shown to increase survival (Diel *et al*, 2008).

We, and others, have shown that carcinoma-associated fibroblasts derived from human prostate tumours can promote tumourigenesis (Olumi *et al*, 1999; Barclay *et al*, 2005; Franco *et al*, 2011; Kiskowski *et al*, 2011). We have also demonstrated that the stromal phenotype in a tumour can be used as a basis for patient disease-progress prognostication (Ayala *et al*, 2003; Yanagisawa *et al*, 2007). Although some of the pathways underlying the ability of cancer stroma to regulate tumourigenesis have been elucidated (Ao *et al*, 2006, 2007; He *et al*, 2007), the situation is complex and many interactions remain to be explored (Bierie and Moses, 2006), especially with regard to the progression of the carcinoma towards either stromagenic (Figure 1 left) or stromal-independent outcomes (Figure 1 right).

In this paper we introduce an Evolutionary Game Theory (EGT) model that studies the evolution of three different cell populations over time: stromal cells, a dependant tumour phenotype capable of co-opting stromal cells to support its growth, and an independent tumour phenotype that does not require microenvironmental support, be it stromal associated or not. This model is then applied to the clinical problem of metastatic prostate cancer.

## The game

In EGT, the behaviour of the players is not assumed to be based on rational payoff maximisation, but it is shaped by trial and error adaptation through natural selection (Smith, 1982). In EGT, a strategy is not a deliberate course of action but a phenotypic trait. The payoff is fitness in the Darwinian sense: more average reproductive success. In this context, interactions between the players are important, be it in a cooperative or competitive manner, as they determine whether it will become a larger share of the population (Sigmund and Nowak, 1999). Fitness is also population dependant, so a given subpopulation might become more (or less) fit if the numbers of a different subpopulation with whom it interacts go up (or down).

Genetic and epigenetic changes can transform the cells in a healthy tissue. One change is to produce individualistic tumour cells that compete for space and resources (Nowell, 1976; Crespi and Summers, 2005; Merlo *et al*, 2006) and which can attract support from other cell types, for example, by replicating developmental scenarios where cell growth is a normal outcome. Under the view of tumours as ecosystems, it is possible to use tools from ecology, such as EGT, to study the evolution of the different cellular populations. EGT has been used to explore various aspects of cancer (Tomlinson and Bodmer, 1997), including glioma progression (Basanta *et al*, 2008, 2011), the influence of the tumour–host interface in colorectal carcinogenesis (Gatenby and Vincent, 2003), the role of phenotypic variability in multiple myeloma (Dingli *et al*, 2009), and the evolution of a number of phenotypic traits such as motility and invasion (Mansury *et al*, 2006; Bellomo *et al*, 2008) or microenvironmental independence (Anderson *et al*, 2009).

As in our previous work (Anderson *et al*, 2009), the model assumes a tumour with two different epithelial phenotypes: tumour cells that depend on the microenvironment for survival (D) and those that are independent of the microenvironment (I). Table 1 shows the payoffs for each cell type when interacting with others. A further assumption is that no other phenotypes are relevant in the context of the game and that spatial considerations will not affect the outcome (Hofbauer and Sigmund, 1998). The payoffs in EGT represent the fitness change resulting from the interaction – a positive change represents an increase in the long-term growth rate of the cell. The payoff values are normalised in the range (0 : 1) so 1 represents the maximum fitness for any given phenotype.

The I cells are relatively independent from the microenvironment and produce their own growth factors (e.g. testosterone) and thus are considered to have a comparatively constant fitness (1-*γ*), where *γ* represents the fitness cost for I cells to be independent, instead of committing those resources to faster proliferation. The D cells rely more on their microenvironment for survival and growth at a fitness cost (*β*) that represents the scarcity of resources or space that I cells can procure themselves. A resource-poor microenvironment would then be characterised by a higher value of *β*. As I cells produce space and shareable growth factors, this model assumes that D cells derive a fitness advantage from their interactions with I cells represented by the variable *ρ*. On the other hand, D cells interacting with other D cells will have a harder time sharing existing microenvironmental resources with other equally dependant cells and thus are assumed to have double the cost 2*β* for relying on the microenvironment for survival and growth and thus have a fitness of 1–2*β*.

A key development from our previous work (Anderson *et al*, 2009) is that we now also consider a stromal population (S) that can interact with the tumour. The stromal compartment is thought to be complicit in tumour progression and represents a potential target for new therapies (Strand *et al*, 2010) as well as possibly an under-recognised one for current therapies such as androgen ablation and bisphosphonates. Stromal cells retain the ability to undergo rapid proliferation, but normally are relatively growth quiescent with low rates of proliferation and death. For this reason the fitness benefit derived by stromal cells from the interactions with tumour cells is assumed to be zero. However, under certain circumstances, stromal cells are susceptible to being co-opted by certain tumour phenotypes (much like carcinoma-associated fibroblasts). In this situation, stromal and tumour cells produce factors that stimulate each other's growth in a mutualistic manner. For the EGT model this is represented by the variable *α* in the payoff table. A low *α* represents tumours in which the stroma cannot be co-opted. There are only four variables in the model,which is the minimum necessity to consider how the costs and benefits of either relaying on the stroma and the stromal cells (*α*, *β*, *ρ*) or on the other hand being independent (*γ*) affect the outcome of prostate cancer.

If p^I^_t_ is the proportion of I cells at a given time *t* and p^D^_t_ the proportion of D cells, then the absolute fitness of each cell population (*W(*S*)*, *W(*I*)*, *W(*D*)*) will be given by the following expressions:



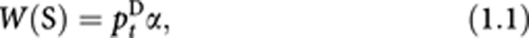





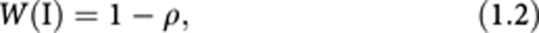





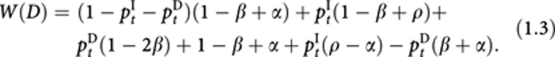



The average fitness 
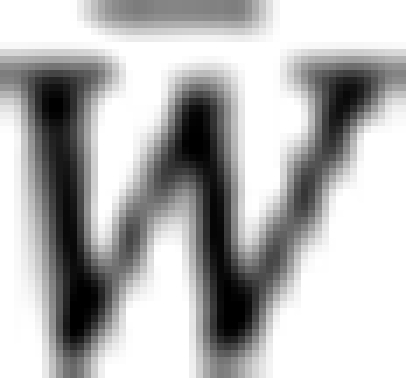
 of the population is given by:



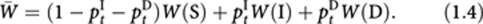



From these expressions it is possible to derive the discrete replicator equations that describe how they change over time (Smith, 1982). The proportion of a cellular population in the model at a given time *t* will depend not only on its own fitness (W) but also on the fitness of the other cell populations. If the fitness of a phenotype X, W(X) is higher than the average fitness of all the phenotypes combined <W> then the proportion of that phenotype will increase during the next time step, for as long as the reasons that keep the phenotype relatively fit remain. The replicator equations are:



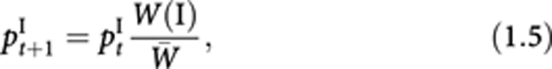





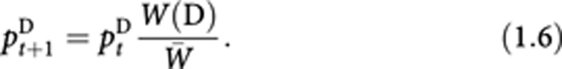



## Results

One may apply the replicator equations described in the previous section to study the temporal evolution of the different populations in a number of scenarios. These scenarios are characterised by the four variables of the model: *α*, the mutual benefit between D cells and co-opted stroma; *ρ*, the benefit that D cells derive from coexisting with I cells (which can produce growth factors and space); *β*, the fitness cost of relying on a microenvironment that might not be able to produce resources that I cells can produce on its own and *γ*, the cost that I cells have to incur to become independent from the microenvironment.

We considered three scenarios describing rich, neutral and poor environments (assigned as three values of the fitness cost of dependence, *β*=0.2, 0.5 and 0.8). In each case, the replicator equation was iterated 20 times from an initial condition assuming that both tumour populations represented a very small proportion, each 10^−4^ of the population, with the rest being stromal cells (S). We assumed that each of the tumour populations is equally likely of arising from the normal epithelial population (not modelled) as there is no conclusive evidence of one of the abnormal ones necessarily arising from the other (e.g.: I cells appearing as mutant D cells or *vice versa*). Different initial conditions yield similar results although the time required to reach equilibrium depends on the proportion of tumour cells in the population. We chose 20 time steps, as it appears to be sufficient for most simulations to reach stability (see also the Appendix for an actual stability analysis).

**Figure 2:**
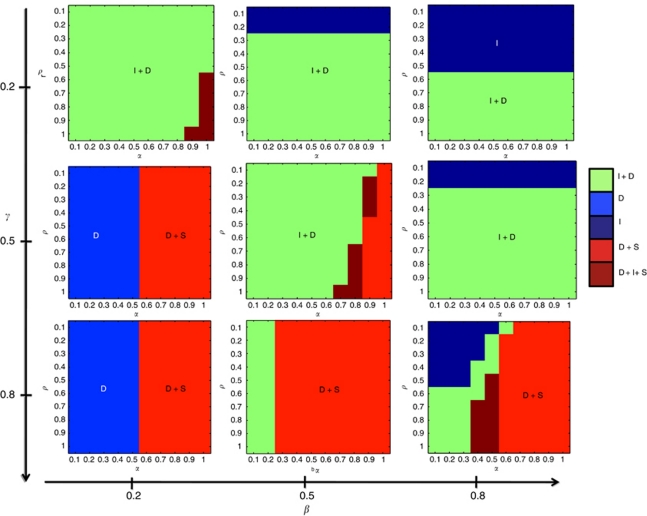
Outcomes from the replicator equations under a range of costs associated with S, I and D phenotypes. Each box represents the outcome of the replicator equation in which the specific values of *α* and *ρ* are varied (from 0.1 to 1). We are assuming three types of scenarios in terms of availability of resources and space: from rich (left column, characterised by *β*=0.2) to medium (centre column, *β*=0.5) to poor (right column, *β*=0.8). We have also hypothethised three different I phenotypes, from very fit (upper row, with *γ*=0.2) to medium (centre row, *γ*=0.5) and unfit (lower row, *γ*=0.8).

The first row in Figure 2 shows the outcomes from three scenarios characterised by different costs of dependence on the microenvironment with a baseline I phenotype cost set to *γ*=0.2. When the microenvironment is rich in resources (*β*=0.2), the main outcomes are either coexistence of tumour phenotypes or, when *α* or *ρ* is high (increasing the fitness of the D population), coexistence of the three phenotypes. The second row shows the outcomes from three scenarios characterised by different costs of relying on the microenvironment with an I phenotype with *γ*=0.5. When the microenvironment is rich in resources the main outcomes are either dominance of D phenotypes or, when *γ* is sufficiently high, coexistence of D and S.

When resources are neither scarce nor plentiful the main outcomes are coexistence of I and D if *α* is sufficiently small or coexistence of I and stroma if *α* is high enough. Interestingly for some values of *α* (from medium to intermediate-high), the outcome tends to be coexistence of the three phenotypes. For environments poor in resources (*β*=0.8) the main outcomes are driven by *ρ*, with low values of *ρ* (benefit for D cells coexisting with I cells) leading to dominance of I phenotypes and higher ones leading to coexistence of both tumour phenotypes.

The third row shows the outcomes from three scenarios characterised by different costs of relying on the microenvironment with an I phenotype with *γ*=0.8, representing a relatively unfit I population. When the microenvironment is rich in resources the main outcomes are either dominance of D phenotypes or, when *α* is sufficiently high, coexistence of D and S. When resources are neither scarce nor plentiful the main outcomes are coexistence of I and D if *α* is sufficiently small or coexistence of D and S if *α* is high enough. Perhaps the most dynamically interesting and biologically relevant simulation is when the microenvironment is poor in resources (*β*=0.8) and the cost of being independent is high (*γ*=0.8) as shown in the bottom right corner of Figure 2.

If both *ρ* and *α* are low then the tumour will be dominated by I phenotypes. If *α* is 0.5 or below, a sufficiently high value of *ρ* leads to tumours that contain both I and D cells. When *α* is higher than 0.5 the cooperation between stroma and D results in the extinction of I cells. Coexistence between the three phenotypes occurs when *ρ* is high (which helps the D cells) and *α* is below 0.5 but not too low (over 0.3), which promotes cooperation between D and stromal cells without driving I cells to extinction.

**Figure 3:**
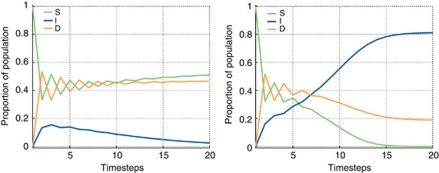
Left: Stromagenic tumour with coexistence of D and stromal phenotypes. In this example the stromal population derives a benefit from its interaction with the D cells. As a result the I population is driven to extinction. The initial proportion of I and D cells is 10^−4^ with the rest being stromal cells. The parameters characterising the game were *α*=0.5, *β*=0.7, *ρ*=0.1 and *γ*=0.8. Right: Stromal-independent tumour with dominance of I phenotypes. In this example the stromal population derives a benefit from its interaction with the D cells, but both populations are still outcompeted by the I cells. As a result the stromal population is driven to extinction and the D population to irrelevancy. The initial proportion of I and D cells is 10^4^ with the rest being stromal cells. The parameters characterising the game were *α*=0.5, *β*=0.7, *ρ*=0.1 and *γ*=0.75.

As the emergence of stromagenic tumours *vs* those independent of stroma is of great interest, the dynamics of these outcomes were explored in more detail. Figure 3 shows the time evolution of the replicator equation for two specific examples. The figure shows how small changes in the fitness of I (as determined by *γ*) or D phenotypes (as determined by *β*, *ρ* and *α*) could result in large changes in the population dynamics leading to fundamentally different outcomes. Left panel: a sufficiently high value of *α* and low value of *ρ* means that D cells derive a much higher benefit from their cooperation with stromal cells than from their interactions with I cells. Given the low general fitness of the I cells (with *γ*=0.8), it is not surprising that they quickly become extinct as the advantage of cooperation sustains, and promotes D and S cells in the tumour. The right panel in the same figure shows a slightly fitter I phenotype (with *γ*=0.75 instead of 0.8); the I population manages to sustain growth and, as it becomes an increasingly larger part of the tumour population, disrupts the already initiated cooperation between D and S cells, resulting in the extinction of the stromal population and a D population that represents a smaller part of the tumour compared with the previous example.

**Figure 4:**
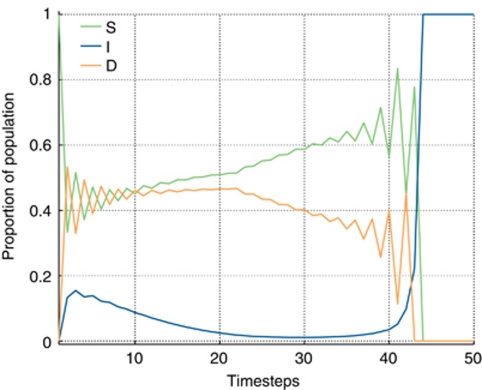
Microenvironmental perturbation leading to a switch from stromagenic to a stromal independent tumour. Starting with the situation described in Figure 3 left, where *α*=0.5, *β*=0.7, *ρ*=0.1 and *γ*=0.8, after iterating the replicator equation for 20 times, *β* was increased by 0.1 to 0.8 to signify a poorer microenvironment. As a result, the D–S cooperation is disrupted and an equilibrium resembling that shown in Figure 3 right is reached.

Changes in the microenvironment can occur during tumour progression both as a result of external intervention (e.g. treatment) and as a result of the tumour itself. Figure 4 illustrates how a dynamic microenvironment can disrupt the outcome and drive the tumour from being stromagenic to stromal independent or *vice versa*. The replicator equations are initially iterated to form a stromagenic tumour (as in the left panel of Figure 3), then after 20 iterations the microenvironment is altered such that it becomes harsher (i.e. increase *β* from 0.8 to 0.9). This results in a destabilisation of the cooperation between the D and S populations, and a state transition to the one exhibiting dominance of the I subpopulation.

## Therapeutic implications

From the results presented in Figure 3, it is clear that small changes in the fitness of the I phenotypes can lead to large-scale changes in populations – even causing transitions from stromal independence to stromagenic tumours. Although both of these tumour types are eventually lethal, it has been postulated that the stromagenic tumours have a longer natural history (Ayala *et al*, 2003, 2011; Strand *et al*, 2010). Further, because of the biological aspects of the different phenotypes, they are likely sensitive to different types of therapy. For example, manipulation of a biological pathway (such as mTOR) would differentially penalise cells which depend more upon intrinsic signalling (I), whereas manipulation of stromal cells or tumour–stroma signalling (such as hormonal therapy for localised disease or a bisphosphonate for bony metastatic disease) would preferentially effect the D cells. In addition, as the steady states depend not only on the parameters (which can be manipulated by therapy) but also on the relative populations, the timing of therapy can drastically change the results.

**Figure 5:**
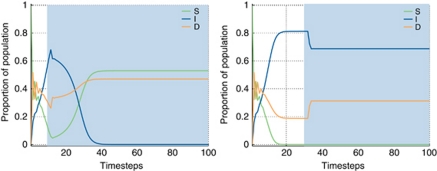
The importance of timing. In all cases we start the simulations with *α*=0.5, *β*=0.7, *ρ*=0.1 and *γ*=0.8. Left we show an early initiation with a therapy directed at stromal-independent cells which effectively destroys the population before it can take over. Right we show the same therapeutic intervention that is initiated at a later time and there is an effect, but it does not change the biology of the tumour fundamentally.

An exploration of a putative biological therapeutic agent with different timing strategies is represented in Figure 5. In the case of this simulated biological therapy (possibly representing an mTOR inhibitor–which preferentially penalises the I population), the end effect of the therapy is strongly influenced by the time of initiation – when initiated early (when there is still competition between I and D), it drastically alters the outcome of the game, whereas late application (after *I* has already dominated) only causes a small shift. This result can be seen as an application of the kairos principle and speaks to the importance of choosing the right therapy at the right time. Testing this same concept in a more stromal-targeted therapy (e.g. hormonal manipulation) did not produce significant differences, suggesting that a short delay in onset is of less importance in this therapeutic strategy. This result does not suggest that early *vs* late hormone therapy is meaningless – in fact it has been shown that early hormone therapy can slightly increase overall survival, but instead that the timing will not effect the overall outcome of the game.

**Figure 6:**
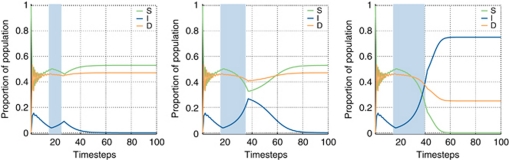
Hormone therapy. In all cases we start the simulations with *α*=0.5, *β*=0.7, *ρ*=0.1 and *γ*=0.8. Left initiation at timestep 15 with a short duration pulse of 10 timesteps of ‘stromal-directed’ therapy shows a very minimal effect and no change in the steady state. Centre initiation at timestep 15 with a medium-duration pulse of 20 timesteps of ‘stromal-directed’ therapy shows a substantial effect that slowly returns to the initial steady state. Right initiation at timestep 15 with a long duration pulse of 25 timesteps of ‘stromal-directed’ therapy shows a substantial effect that changes the overall steady state and effectively changes the biology of the tumour recapitulating the emergence of resistance to therapy.

For testing a different therapeutic strategy, the duration of ‘stromal-directed’ therapy is shown in Figure 6. Here, we hypothesise that the application of such a therapy would primarily effect the benefit D cells receive from the interaction with S cells (i.e. *α* will be reduced). This results in a shift in the opposite direction from the prior therapy – from D to I. Further, we see that the duration of therapy strongly affects the end result of the game. The final result (Figure 6C) recapitulates the clinical reality of evolution of resistance to hormonal therapy (e.g. castrate-resistant prostate cancer).

## Discussion

Although many nascent prostate tumours never become life threatening, those that do can use two different and distinct routes: either becoming microenvironmentally independent (representing a small cell prostate cancer-type scenario) or by co-opting certain stromal cells in order to sustain tumour progression (as happens in bony metastatic disease). We present the simplest model that abstracts key aspects of prostate cancer evolutionary dynamics, including progression towards lethal outcomes that can be either stromagenic (resulting from mutualistic interactions between the tumour and certain stromal cells) or stromal independent. These outcomes reflecting evolutionary changes of the genetically unstable, heterogenous tumour cell population, are influenced by the interactions both between the different populations (I, S and D) and with their microenvironment. Primary tumours are likely to contain areas that are stromagenic as well as other areas that are stromal independent, which would make application of the therapeutical message of this model less relevant. On the other hand, secondary sites are thought to represent clonal populations originating from a specific (either stromagenic or stromal independent) area of the primary tumour (Navin *et al*, 2011); therefore, extrapolating to the metastatic situation is more appropriate.

We have previously that demonstrated (Anderson *et al*, 2009) dominance of D phenotypes happens naturally in a microenvironment rich in resources whereas resource-poor microenvironments tend to select for I cells. These results are further validated by this model: regardless of the absolute fitness of the I phenotype (as given by *γ*), an increase in the proportion of I cells is observed as the microenvironment becomes resource-poor (as given by *β*, right column of Figure 2). Stroma and its interactions with the tumour have a tremendous impact on the phenotypic composition of the tumour. In those cases in which *α* is sufficiently high, denoting stroma that can be supported by the tumour, the cooperation between D and S can push the I population towards extinction (see Figure 3 left panel). Conversely, this can be described as tumour cells that are particularly effective at stimulating stromal support – the key is the interactive process, not so much the cell's absolute behaviour.

On the other hand, when *ρ* is sufficiently high, the ensuing mutualistic relationship between D and I can lead to dominance of the tumour phenotypes at the expense of S, even when the D cells can derive some benefit from their interactions with the stroma (see Figure 3 right panel). The least frequent of the outcomes, coexistence of the three phenotypes, depends on values of *α* being high enough to promote the collaboration between D and stromal cells, but not so high that I cells are driven to extinction. Higher values of *ρ* promote polyclonal tumours as they allow D cells to coexist with I cells. Thus, those tumours in which the D cells can benefit from the factors produced by I cells and still cooperate with the stroma are more likely to sustain the three phenotypes in an evolutionarily stable strategy.

To combat the emergence of castrate resistance (and side effects) in the treatment of metastatic prostate cancer, many physicians have adopted non-standard dosing schedules for their androgen ablation therapies. A recent study suggested that either using the patient's measured testosterone levels as a guide, or using an intermittent schedule as opposed to constant dosing (a calendar schedule) slowed the onset of castrate resistance (Blumberg *et al*, 2011). Although this study has shown that alternative schedules can provide benefit, there is, as of yet, no standard of care for this. To explore this question, simulations were run with differing times of initiation and duration of stromal-directed therapy. The results of the game given different durations of stromal-directed therapy show significant, fundamental differences in outcome with differing schedules. Initiation with short duration of treatment (Figure 6A) results in no appreciable change in the game. Initiation for long duration (Figure 6C), as expected, shows the evolution of castrate resistance and a phase transition to a new steady state dominated by I cells, which will then be insensitive to further manipulation with this therapy, requiring a strategy change. There is, however, an optimal duration (Figure 6B) where the D cells are reduced without an irreversible increase in the I cells. When the therapy is taken off, the levels begin to return to the original D-dominated steady state, a situation where ‘stromal-directed’ therapy will work again.

These results, taken together, suggest that different therapies are likely of different value to the two major tumour types: D tumours are likely best treated with stromal manipulation (e.g. hormonal therapy) whereas I tumours would be better treated initially with a biological agent such as an mTOR inhibitor.

Regardless of timing and schedule, the emergence of castrate resistance is largely unavoidable given a long enough time frame, and these results suggest that when this emergency does occur, a treatment strategy change is in order. These sentiments are echoed in the recent literature which suggests that the addition of mTOR inhibitors to hormone therapy after the onset of castrate resistance (Schayowitz *et al*, 2010) could hold promise.

## Conclusions

In this paper we have introduced a minimal mathematical model to understand how the stromal cells can influence prostate cancer progression. The results presented here are qualitative (rather than quantitative), but highlight the importance of understanding tumour–stroma interactions in driving not only tumour outcomes (whether malignant or not) but also their impact on potentially new therapeutic approaches. We have explored vast parts of the parameter space, so we are confident that these results capture the essence of our prostate cancer model. We made an assumption that the biology of metastatic sites is related to the original biology of the primary tumour. While this assumption carries with it many other assumptions, it is the one that underlies the majority of clinical decision-making and biological extrapolation in prostate cancer.

Our model provides a theoretical framework to understand clinical observations. It is not intended to provide qualitative recommendations for timing and duration of therapy (limited by the arbitrary nature of the time variable in this model). However, this model suggests a number of testable hypotheses. It highlights the importance of the stromal cells in selecting for specific tumour cell phenotypes. It shows that stromagenic tumours are possible (as shown by the results where the steady state is made of S and D types). Importantly the results suggest that coexistence of microenvironmental dependant cells and the stroma is not always robust, and that changes like those resulting from a targeted therapy could transform the tumour into the more aggressive stromal-independent kind.

The power of an abstract model such as this is not necessarily the direct interpretation of results, as many of the parameters tested are, as of yet, not biologically testable or actionable – but instead the method itself for testing new therapeutic strategies. In the clinical paradigm of metastatic prostate cancer, which has recently been muddied with many new agents coming to the arena, it has become ever more important to develop rational strategies to test new sequence and timing regimens. For example, it may be that the time-honoured standard of hormonal manipulation until failure is no longer the best strategy – but how can we ethically and rationally integrate these new therapies without some guidance? The results of this model suggest that with the multi-disciplinary effort of biologists, clinicians and theoreticians, rational strategies can be suggested from the morass of heretofore untested permutations of these new agents. We do not believe that the results presented in this paper are the final answer to the prostate cancer question, but instead hope that they begin conversations between disciplines and stimulate new kinds of questions.

## Figures and Tables

**Figure 1:**
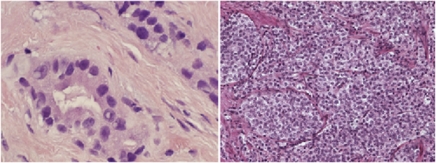
Lethal prostate cancer phenotypes. On the left we show a glandular tumour with abundant reactive stroma (stromogenic carcinoma). The image on the right is a poorly differentiated cancer without intervening reactive stroma (stroma-independent tumour).

**Table 1:** Payoff table that represents the interactions between the three cell types considered in the model

	**S**	**D**	**I**
S	0	*α*	0
D	1+*α*–*β*	1−2*β*	1−*β*+*ρ*
I	1−*γ*	1−*γ*	1−*γ*

The fitness of each of the phenotypes (S, Stroma; D, microenvironmentally dependent; I,microenvironmentally independent) depends on the interactions with other phenotypes and the values of the costs and benefits resulting from these interactions. These costs and benefits are: *α* (benefit derived from the cooperation between a S cell and a D cell), *γ* (cost of being microenvironmentally independent), *β* (cost of extracting resources from the microenvironment and *ρ* (benefit derived by D from paracrine growth factors produced by I cells.

## APPENDIX

### Stability analysis

In this appendix, we will describe the steady-state solutions obtained from analysing the evolutionary stable strategies (ESS) (Smith, 1982). These can be easily obtained by assuming that if a polyclonal tumour was in equilibrium, then the fitness of each of the three coexisting phenotypes should be equivalent (Bishop and Cannings, 1978). Assuming that p and p′ are the proportions of the population made of *I* and *D* cells (with 1–p–p′ the proportion of *S* cells).

If *W*(I)=*W*(D)=*W*(S) then

also

which means that

The ESS for polyclonal tumours that consist of only two phenotypes or those that are monoclonal can be studied in a similar manner by assuming that one or two of the population will not exist in the steady state. Assuming that the I population goes eventually extinct, then the steady state would be a tumour with *D* and stromal cells. If ***W***(**D**) = ***W***(**S**) then

If the equilibrium is between tumour phenotypes (*W*(D)=*W*(I)) then

## References

[bib1] Anderson AR, Hassanein M, Branch KM, Lu J, Lobdell NA, Maier J, Basanta D, Weidow B, Narasanna A, Arteaga CL, Reynolds AB, Quaranta V, Estrada L, Weaver AM (2009) Microenvironmental independence associated with tumor progression. Cancer Res 69(22): 8797–8806 19887618 10.1158/0008-5472.CAN-09-0437 PMC2783510

[bib2] Ao M, Franco OE, Park D, Raman D, Williams K, Hayward SW (2007) Cross-talk between paracrine-acting cytokine and chemokine pathways promotes malignancy in benign human prostatic epithelium. Cancer Res 67(9): 4244–4253 17483336 10.1158/0008-5472.CAN-06-3946

[bib3] Ao M, Williams K, Bhowmick NA, Hayward SW (2006) Transforming growth factor-promotes invasion in tumorigenic but not in nontumorigenic human prostatic epithelial cells. Cancer Res 66(16): 8007–8016 16912176 10.1158/0008-5472.CAN-05-4451 PMC4067141

[bib4] Ayala GE, Muezzinoglu B, Hammerich KH, Frolov A, Liu H, Scardino PT, Li R, Sayeeduddin M, Ittmann MM, Kadmon D, Miles BJ, Wheeler TM, Rowley DR (2011) Determining prostate cancer-specific death through quantification of stromogenic carcinoma area in prostatectomy specimens. Am J Pathol 178(1): 79–87 21224046 10.1016/j.ajpath.2010.09.042 PMC3069909

[bib5] Ayala G, Tuxhorn JA, Wheeler TM, Frolov A, Scardino PT, Ohori M, Wheeler M, Spitler J, Rowley DR (2003) Reactive stroma as a predictor of biochemical-free recurrence in prostate cancer. Clin Cancer Res 9(13): 4792–4801 14581350

[bib6] Balkwill F (2009) Tumour necrosis factor and cancer. Nat Rev Cancer 9(5): 361–371 19343034 10.1038/nrc2628

[bib7] Barclay WW, Woodruff RD, Hall MC, Cramer SD (2005) A system for studying epithelial-stromal interactions reveals distinct inductive abilities of stromal cells from benign prostatic hyperplasia and prostate cancer. Endocrinology 146(1): 13–18 15471963 10.1210/en.2004-1123 PMC3033046

[bib8] Basanta D, Scott JG, Rockne R, Swanson KR, Anderson AR (2011) The role of IDH1 mutated tumour cells in secondary glioblastomas: an evolutionary game theoretical view. Phys Biol 8(1): 015016 21301070 10.1088/1478-3975/8/1/015016 PMC3166886

[bib9] Basanta D, Simon M, Hatzikirou H, Deutsch A (2008) Evolutionary game theory elucidates the role of glycolysis in glioma progression and invasion. Cell Prolif 41(6): 980–987 19040573 10.1111/j.1365-2184.2008.00563.x PMC6495695

[bib10] Bellomo N, Chaplain M, De Angelis E (2008) Selected Topics in Cancer Modeling. Genesis, evolution immune competition, and therapy, Birkhauser. ISBN: 0817647120, 9780817647124

[bib11] Bierie B, Moses HL (2006) Tumour microenvironment: TGFbeta: the molecular Jekyll and Hyde of cancer. Nat Rev Cancer 6(7): 506–520 16794634 10.1038/nrc1926

[bib12] Bishop DT, Cannings C (1978) A generalized war of attrition. J Theor Biol 70(1): 85–124 564432 10.1016/0022-5193(78)90304-1

[bib13] Blumberg JM, Kwon EO, Cheetham TC, Niu F, Shapiro CE, Pacificar J, Loo RK, Williams SG, Chien GW (2011) Early development of castrate resistance varies with different dosing regimens of luteinizing hormone releasing hormone agonist in primary hormonal therapy for prostate cancer. Urology 77(2): 412–416 21111460 10.1016/j.urology.2010.08.037

[bib14] Crespi B, Summers K (2005) Evolutionary biology of cancer. Trends Ecol Evol 20(10): 545–552 16701433 10.1016/j.tree.2005.07.007

[bib15] De Marzo AM, Platz EA, Sutcliffe S, Xu J, Grönberg H, Drake CG, Nakai Y, Isaacs WB, Nelson WG (2007) Inflammation in prostate carcinogenesis. Nat Rev Cancer 7(4): 256–269 17384581 10.1038/nrc2090 PMC3552388

[bib16] Diel IJ, Jaschke A, Solomayer EF, Gollan C, Bastert G, Sohn C, Schuetz F (2008) Adjuvant oral clodronate improves the overall survival of primary breast cancer patients with micrometastases to the bone marrow: a long-term follow-up. Ann Oncol 19(12): 2007–2011 18664560 10.1093/annonc/mdn429 PMC2733118

[bib17] Dingli D, Chalub FA, Santos FC, Van Segbroeck S, Pacheco JM (2009) Cancer phenotype as the outcome of an evolutionary game between normal and malignant cells. Br J Cancer 101(7): 1130–1136 19724279 10.1038/sj.bjc.6605288 PMC2768082

[bib18] Franco OE, Jiang M, Strand DW, Peacock J, Fernandez S, Jackson II RS, Revelo MP, Bhowmick NA, Hayward SW (2011) Altered TGF-*β* signaling in a subpopulation of human stromal cells promotes prostatic carcinogenesis. Cancer Res 71(4): 1272–1281 21303979 10.1158/0008-5472.CAN-10-3142 PMC3076790

[bib19] Gallo M, De Luca A, Lamura L, Normanno N (2011) Zoledronic acid blocks the interaction between mesenchymal stem cells and breast cancer cells: implications for adjuvant therapy of breast cancer. Ann Oncol; e-pub ahead of print 12 May 2011; doi: 10.1093/annonc/mdr159 10.1093/annonc/mdr159 21551002

[bib20] Gatenby RA, Vincent TL (2003) An evolutionary model of carcinogenesis. Cancer Res 63(19): 6212–6220 14559806

[bib21] He Y, Franco OE, Jiang M, Williams K, Love HD, Coleman IM, Nelson PS, Hayward SW (2007) Tissue-specific consequences of cyclin D1 overexpression in prostate cancer progression. Cancer Res 67(17): 8188–8197 17804732 10.1158/0008-5472.CAN-07-0418

[bib22] Hofbauer J, Sigmund K (1998) Evolutionary Games and Population Dynamics. Cambridge University Press: Cambridge

[bib23] Kiskowski MA, Jackson II RS, Banerjee J, Li X, Kang M, Iturregui JM, Franco OE, Hayward SW, Bhowmick NA (2011) Role for stromal heterogeneity in prostate tumorigenesis. Cancer Res 71(10): 3459–3470 21444670 10.1158/0008-5472.CAN-10-2999 PMC3096737

[bib24] Kundu JK, Surh YJ (2008) Inflammation: gearing the journey to cancer. Mutat Res 659(1-2): 15–30 18485806 10.1016/j.mrrev.2008.03.002

[bib25] Lynch CC, Hikosaka A, Acuff HB, Martin MD, Kawai N, Singh RK, Vargo-Gogola TC, Begtrup JL, Peterson TE, Fingleton B, Shirai T, Matrisian LM, Futakuchi M (2005) MMP-7 promotes prostate cancer-induced osteolysis via the solubilization of RANKL. Cancer Cell 7(5): 485–496 15894268 10.1016/j.ccr.2005.04.013

[bib26] Mansury Y, Diggory M, Deisboeck TS (2006) Evolutionary game theory in an agent-based brain tumor model: exploring the ‘Genotype-Phenotype’ link. Bull Math Biol 238(1): 146–156 10.1016/j.jtbi.2005.05.027 16081108

[bib27] Mantovani A, Allavena P, Sica A, Balkwill F (2008) Cancer-related inflammation. Nature 454(7203): 436–444 18650914 10.1038/nature07205

[bib28] Merlo LM, Pepper JW, Reid BJ, Maley CC (2006) Cancer as an evolutionary and ecological process. Nat Rev Cancer 6(12): 924–935 17109012 10.1038/nrc2013

[bib29] Navin N, Kendall J, Troge J, Andrews P, Rodgers L, McIndoo J, Cook K, Stepansky A, Levy D, Esposito D, Muthuswamy L, Krasnitz A, McCombie WR, Hicks J, Wigler M (2011) Tumour evolution inferred by single-cell sequencing. Nature 472(7341): 90–94 21399628 10.1038/nature09807 PMC4504184

[bib30] Nowell P (1976) The clonal evolution of tumor cell populations. Science 194(4260): 23–28 959840 10.1126/science.959840

[bib31] Olumi AF, Grossfeld GD, Hayward SW, Carroll PR, Tlsty TD, Cunha GR (1999) Carcinoma-associated fibroblasts direct tumor progression of initiated human prostatic epithelium. Cancer Res 59(19): 5002–5011 10519415 10.1186/bcr138 PMC3300837

[bib32] Reedy J (1975) Galen on cancer and related diseases. Clio Med 10(3): 227–238 50913

[bib33] Schayowitz A, Sabnis G, Goloubeva O, Njar VC, Brodie AM (2010) Prolonging hormone sensitivity in prostate cancer xenografts through dual inhibition of AR and mTOR. Br J Cancer 103(7): 1001–1007 20842117 10.1038/sj.bjc.6605882 PMC2965879

[bib34] Schetter AJ, Heegaard NH, Harris CC (2010) Inflammation and cancer: interweaving microRNA, free radical, cytokine and p53 pathways. Carcinogenesis 31(1): 37–49 19955394 10.1093/carcin/bgp272 PMC2802675

[bib35] Schor SL, Schor AM, Howell A, Crowther D (1987) Hypothesis: persistent expression of fetal phenotypic characteristics by fibroblasts is associated with an increased susceptibility to neoplastic disease. Exp Cell Biol 55(1): 11–17 3569635 10.1159/000163389

[bib36] Schor SL, Schor AM, Rushton G (1988) Fibroblasts from cancer patients display a mixture of both foetal and adult-like phenotypic characteristics. J Cell Sci 90(Pt 3): 401–407 3253290 10.1242/jcs.90.3.401

[bib37] Schor SL, Schor AM (1987) Clonal heterogeneity in fibroblast phenotype: implications for the control of epithelial-mesenchymal interactions. BioEssays 7(5): 200–204 3325049 10.1002/bies.950070503

[bib38] Sigmund K, Nowak MA (1999) Evolutionary game theory. Curr Biol 9(14): R503–R505 10576907 10.1016/s0960-9822(99)80321-2

[bib39] Smith JM (1982) Evolution and the Theory of Games. Cambridge University Press: Cambridge, UK

[bib40] Strand DW, Franco OE, Basanta D, Anderson AR, Hayward SW (2010) Perspectives on tissue interactions in development and disease. Curr Mol Med 10(1): 95–112 20205682 10.2174/156652410791065363 PMC4195241

[bib41] Tomlinson IP, Bodmer WF (1997) Modelling the consequences of interactions between tumour cells. Br J Cancer 75(2): 157–160 9010019 10.1038/bjc.1997.26 PMC2063276

[bib42] Yanagisawa N, Li R, Rowley D, Liu H, Kadmon D, Miles BJ, Wheeler TM, Ayala GE (2007) Stromogenic prostatic carcinoma pattern (carcinomas with reactive stromal grade 3) in needle biopsies predicts biochemical recurrence-free survival in patients after radical prostatectomy. Hum Pathol 38(11): 1611–1620 17868773 10.1016/j.humpath.2007.04.008

